# An Ensemble Classifier for Eukaryotic Protein Subcellular Location Prediction Using Gene Ontology Categories and Amino Acid Hydrophobicity

**DOI:** 10.1371/journal.pone.0031057

**Published:** 2012-01-30

**Authors:** Liqi Li, Yuan Zhang, Lingyun Zou, Changqing Li, Bo Yu, Xiaoqi Zheng, Yue Zhou

**Affiliations:** 1 Department of Orthopedics, Xinqiao Hospital, Third Military Medical University, Chongqing, China; 2 Department of Microbiology, College of Basic Medical Sciences, Third Military Medical University, Chongqing, China; 3 Department of Orthopedics, Yichun People's Hospital, Yichun, China; 4 Department of Mathematics, Shanghai Normal University, Shanghai, China; 5 Scientific Computing Key Laboratory of Shanghai Universities, Shanghai, China; Dana-Farber Cancer Institute, United States of America

## Abstract

With the rapid increase of protein sequences in the post-genomic age, it is challenging to develop accurate and automated methods for reliably and quickly predicting their subcellular localizations. Till now, many efforts have been tried, but most of which used only a single algorithm. In this paper, we proposed an ensemble classifier of KNN (*k*-nearest neighbor) and SVM (support vector machine) algorithms to predict the subcellular localization of eukaryotic proteins based on a voting system. The overall prediction accuracies by the *one-versus-one* strategy are 78.17%, 89.94% and 75.55% for three benchmark datasets of eukaryotic proteins. The improved prediction accuracies reveal that GO annotations and hydrophobicity of amino acids help to predict subcellular locations of eukaryotic proteins.

## Introduction

Researches on subcellular location of proteins are important for elucidating their functions involved in various cellular processes, as well as in understanding some disease mechanisms and developing novel drugs. Since experimental determinations of the localization are time-consuming, tedious and costly, especially for the rapid accumulation of protein sequences, it is highly desirable to develop effective computational methods for accurately and quickly predicting their subcellular attributes.

In the past few years, many computational methods have been developed for this purpose [1], [2], [3], [4]. These methods can be divided into two main categories [5]. Methods in the first category are based on the observation that amino acid compositions of extracellular and intracellular proteins are significantly different [6]. Along this line, many computational approaches based on amino acid composition, dipeptide composition [7] and gapped amino acid pairs [8] were proposed. Meanwhile, to incorporate more sequence information, many other features were incorporated, such as amphiphility of amino acids [9], functional domain composition [10], psi-blast profile [11], [12] and so on. Methods in the second category are based on a certain sorting signals [13], [14], including signal peptides, chloroplast transit peptides and mitochondrial targeting peptides. For example, Emanuelsson et al. [14] provided detailed instructions for the use of SignalP and ChloroP in prediction of cleavage sites for secretory pathway signal peptides and chloroplast transit peptides. However, the reliability of these methods is highly dependent on protein N-terminal sequence assignments, and the molecular mechanisms related to sorting signals are rather complex and not interpreted clearly.

Not only protein sequence information but also prediction algorithms could affect the accuracy of the subcellular localization prediction. So far, many computational techniques, such as the hidden Markov models (HMM) [15], [16], neural network [17], *K*-nearest neighbor (KNN) [18] and support vector machine (SVM) [5], [19] were introduced for the prediction of protein subcellular localization. However, most of the current predictors are based on a single theory which could have its own inherent defects, so their predictions are not satisfactory. For example, the number of parameters that need to be evaluated in an HMM is large [20]. The neural network can suffer from multiple local minima [21]. Besides, quite a few ensemble classifiers [7], [22], [23] for prediction of protein subcellular localizations have been proposed. However, many of the ensemble classifiers were actually engineered only by a single algorithm, such as the fuzzy KNN [7], KNN [22], and Bayesian [23]. Other ensemble classifiers, such as CE-PLoc [24] and the KNN-SVM ensemble classifier proposed by Zhang [25], were engineered by different algorithms, mostly including SVM and KNN. Along this line, an ensemble classifier making use of the classical SVM and KNN algorithms was developed in this article to predict subcellular localization of eukaryotic proteins.

We apply our method to three widely used eukaryotic protein datasets. By the jackknife cross-validation test [26], [27], [28], [29], the ensemble classifier shows high accuracies and may play an important complementary role to existing methods.

## Materials and Methods

### 1. Datasets

In order to evaluate the performance of the proposed method and compare it with current methods, we introduced three widely used datasets into this study. The first dataset was constructed by Chou [30]. This dataset (denoted as iLoc8897) consists of 8,897 locative protein sequences (7,766 different proteins), which divided into 22 subcellular locations. Among the 7,766 different eukaryotic proteins, 6,687 belong to one subcellular location, 1,029 to two locations, 48 to three locations, and 2 to four locations. None of the proteins has ≥25% sequence identity to any other in the same subset. The second benchmark dataset was constructed by Park and Kanehisa [8]. This dataset (denoted as Euk7579) contains 7579 proteins, which are divided into 12 subcellular locations. Proteins in this dataset have the pairwised sequence similarity below 80%. The third dataset was constructed by Shen and Chou [31]. This dataset (denoted as Hum3681) consists of 3,681 locative protein sequences (3,106 different human proteins), which are divided into 14 human subcellular locations. Among the 3,106 different proteins, 2,580 belong to one subcellular location, 480 to two locations, 43 to three locations, and 3 to four locations. None of the proteins has ≥25% sequence identity to any other in the same subcellular location. The detailed information of the three datasets are listed in **Table 1**.

**Table 1:** Three benchmark datasets used to train and test our predictor.

iLoc8897		Euk7579		Hum3681	
Subcellular location	Number of proteins	Subcellular location	Number of proteins	Subcellular location	Number of proteins
Acrosome	14	Chloroplast	671	Centriole	77
Cell membrane	697	Cytoplasm	1241	Cytoplasm	817
Cell wall	49	Cytoskeleton	40	Cytoskeleton	79
Centrosome	96	Endoplasmic reticulum	114	Endosome	24
Chloroplast	385	Extracell	861	Endoplasmic reticulum	229
Cyanelle	79	Golgi apparatus	47	Extracell	385
Cytoplasm	2186	Lysosomal	93	Golgi apparatus	161
Cytoskeleton	139	Mitochondrion	727	Lysosome	77
Endoplasmic reticulum	457	Nucleus	1932	Microsome	24
Endosome	41	Peroxisomal	125	Mitochondrion	364
Extracell	1048	Plasma membrane	1674	Nucleus	1021
Golgi apparatus	254	Vacuolar	54	Peroxisome	47
Hydrogenosome	10	-	-	Plasma membrane	354
Lysosome	57	-	-	Synapse	22
Melanosome	47	-	-	-	-
Microsome	13	-	-	-	-
Mitochondrion	610	-	-	-	-
Nucleus	2320	-	-	-	-
Peroxisome	110	-	-	-	-
Spindle pole body	68	-	-	-	-
Synapse	47	-	-	-	-
Vacuole	170	-	-	-	-
Total	8897	Total	7579	Total	3681

### 2. Gene Ontology

Gene Ontology (GO) is a major bioinformatics initiative. It meets the need for consistent descriptions of gene products in different databases. Gene Ontology database is established on the three criteria: molecular function, cellular component and biological process. It has been developed to manage the overwhelming mass of current biological data from a computational perspective and become a standard tool to annotate gene products for various databases [32], [33]. Accordingly, GO annotation has been being used for diverse sequence-based prediction tasks, such as analyzing the pathogenic gene function with human squamous cell cervical carcinoma [34], mapping molecular responses to xenoestrogens [35], predicting the enzymatic attribute of proteins [36], predicting the transcription factor DNA binding preference [37], and predicting the eukaryotic protein subcellular localization [38]. In particular, the growth of Gene Ontology databases has increased the effectiveness of GO-based features [39]. As a result, Gene Ontology could be used to improve the predictive performance of protein subcellular localization [22], [40].

We downloaded all GO data at ftp://ftp.ebi.ac.uk/pub/databases/GO/goa/UNIPROT/(released on March 15, 2010), and searched the GO terms for all the protein entries in the three datasets. We eliminate those proteins, which have no corresponding GO terms and the number (60, 127 and 4 for the iLoc8897, Euk7579 and Hum3681 datasets) are relatively small compared to the total datasets. We consider this would not have a great influence on its final accuracy. After this step, we got a list of GO terms for each protein entry of the three datasets. For example, the human protein entry “Q9H400” in the Hum3681 dataset corresponds to four GO numbers, i.e., GO: 0005886, GO: 0006955, GO: 0016020 and GO: 0016021, while the protein entry “P81084” in the Euk7579 dataset corresponds to six GO numbers, i.e., GO: 0000166, GO: 0005524, GO: 0006950, GO: 0009507, GO: 0009536 and GO: 0009570. So as to handle these GO numbers efficiently, a compression procedure was proposed to renumber them. For example, all involved GO numbers for the eukaryotic proteins in the Euk7579 dataset are GO: 0000001, GO: 0000002, GO: 0000003, GO: 0000006, GO: 00000009, GO: 0000011, GO: 0000012, …, GO: 0090184. They are renamed as GO_compress: 0000001, GO_compress: 0000002, GO_compress: 0000003, GO_compress: 0000004, GO_compress: 0000005, GO_compress: 0000006, GO_compress: 0000007, ……, GO_compress: 0006533, respectively. When this treatment finished, we got the GO_compress database that contained 6533 numbers. We numbered those data from 1 to 6533. The total numbers of GO terms that appeared for the iLoc8897, Euk7579 and Hum3681 datasets were 7871, 6533 and 5553.

As we know, if we want to describe all possible GO terms for a certain dataset, the simplest way to vector represent a protein was using a binary feature component for a protein. We used value 1 if the corresponding GO number appears and value 0 if it does not appear. For example, the human protein entry “Q8TDM5” in the Hum3681 dataset corresponds to seven GO numbers in the GO database, i.e., GO: 0001669, GO: 0005515, GO: 0005886, GO: 0007155, GO: 0016020, GO: 0031225 and GO: 0031410, which corresponded to GO_compress: 0000212, GO_compress: 0001037, GO_compress: 0001203, GO_compress: 0001722, GO_compress: 0002543, GO_compress: 0003360, GO_compress: 0003398 in the GO_compress database. So the 212^th^, 1037^th^, 1203^rd^, 1722^nd^, 2543^rd^, 3360^th^, and 3398^th^ components of the feature vector were assigned the value 1 and the rest 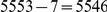 components with the value 0. At last, we transformed the GO terms annotated for each human protein into a 5553-dimension input vector.

### 3. Amphiphilic pseudo amino acid composition

In a protein, the hydrophobicity and hydrophilicity of the native amino acids play an important part in its folding, interior packing, catalytic mechanism, as well as its interaction with other molecules in the environment [41]. Therefore, the two indices may be used to effectively reflect the subcellular locations of proteins. Both the hydrophobicity and hydrophilicity are introduced in the concept of AmPseAAC. As we know, the concept of AmPseAAC proposed by Chou [22] was widely used by many researchers in improving the prediction quality for protein subcellular localization [42], [43]. Following the concept of AmPseAAC, a protein sample could be descripted by a 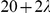 dimensional feature vector, where 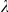 is equal to 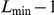, where 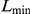 is the length of the shortest protein sequence in the dataset. The 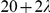 dimensional feature vector for a protein comprises 20 features of the conventional amino acid composition (AAC), and the rest 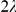 components reflect its sequence-order pattern through the amphiphilic feature. The protein representation is called the “amphiphilic pseudo amino acid composition” or “AmPseAAC” for short. In order to get more local sequence information, we incorporated 400 dipeptide components to the AmPseAAC. Then the new AmPseAAC is constructed and the dimension is increased to 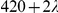, which are 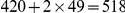, 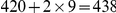, and 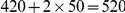 for the iLoc8897, Euk7579 and Hum3681 datasets, respectively. Then we combined the new AmPseAAC and Gene Ontology as the features for protein subcellular localization prediction. As a result, the dimensions of the final input feature vectors are 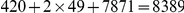, 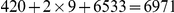, and 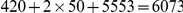 for the iLoc8897, Euk7579 and Hum3681 datasets.

### 4. Feature extraction

**Figure 1:**
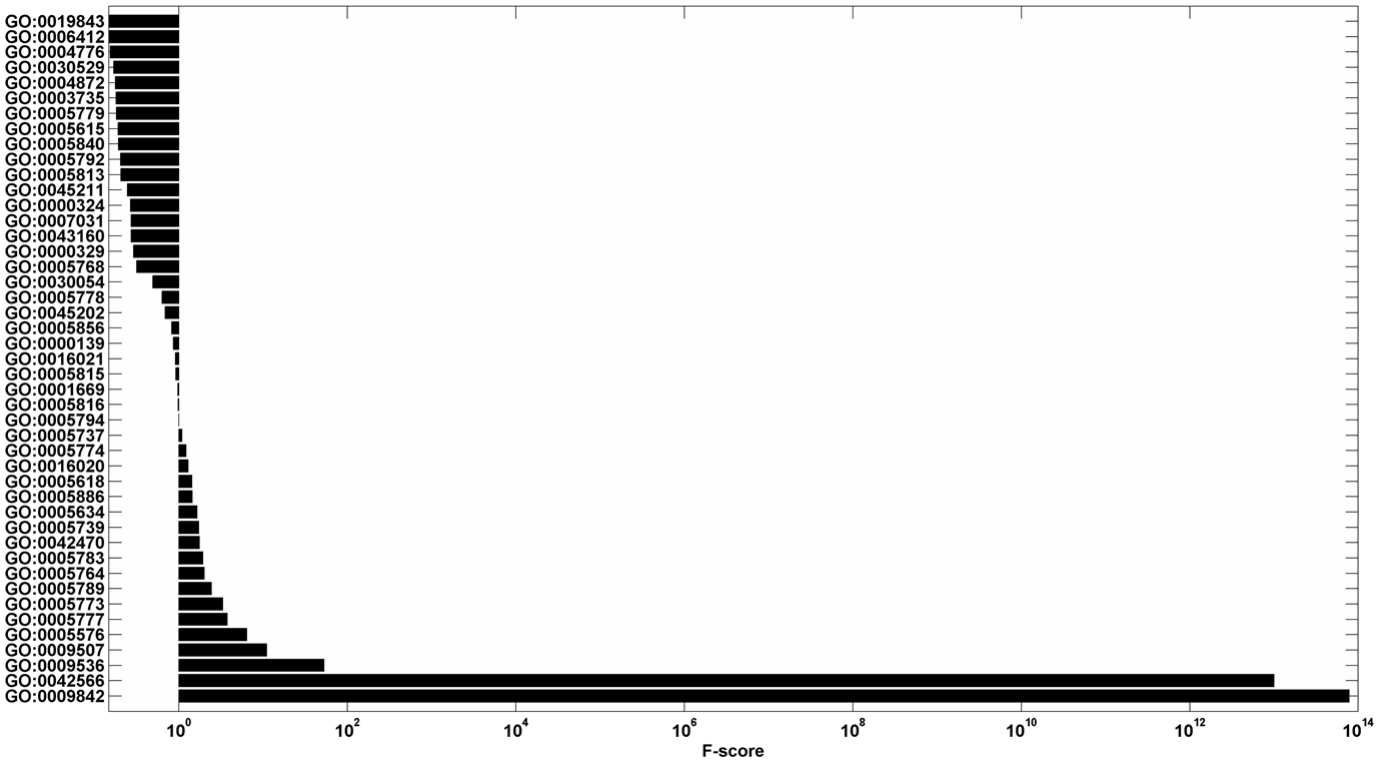
This graph shows the contribution scores of top 45 features on the iLoc8897 dataset.

Due to the limited numbers of learning examples, learning with a small number of features often leads to a better generalization of machine learning algorithms (Occam's razor) [44]. Additionally, with the increase of the dimension of the feature vector, the computational loads for some machine-learning tools, e.g., Support Vector Machine [45] and Neural Network [46], are seriously affected. As a result, we used the “fselect.py” in Libsvm software package to reduce the dimensionality. The fselect.py is a simple python script used F-score to select features. After running the python script, one could get an output file called “.fscore”, in which each feature was given a score to describe the importance of it and all features were sorted by their scores. Then we chose the top features with the highest contribution scores (**Figs. 1** **,** **2** **, and** **3**).

**Figure 2:**
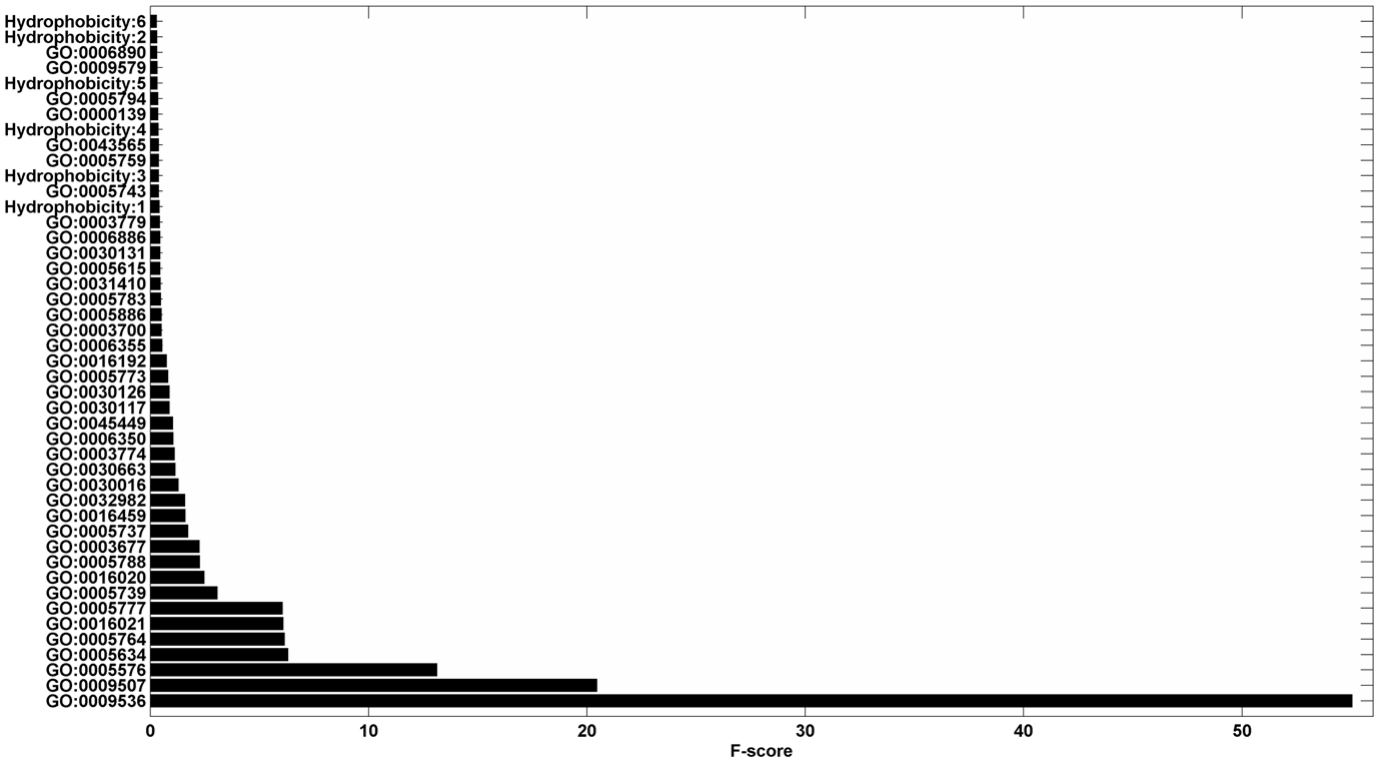
This graph shows the contribution scores of top 45 features on the Euk7579 dataset. Hydrophobicity: 6, 2, 5 … stand for the 6^th^, 2^nd^, 5^th^ … elements in the hydrophobicity vectors respectively.

**Figure 3:**
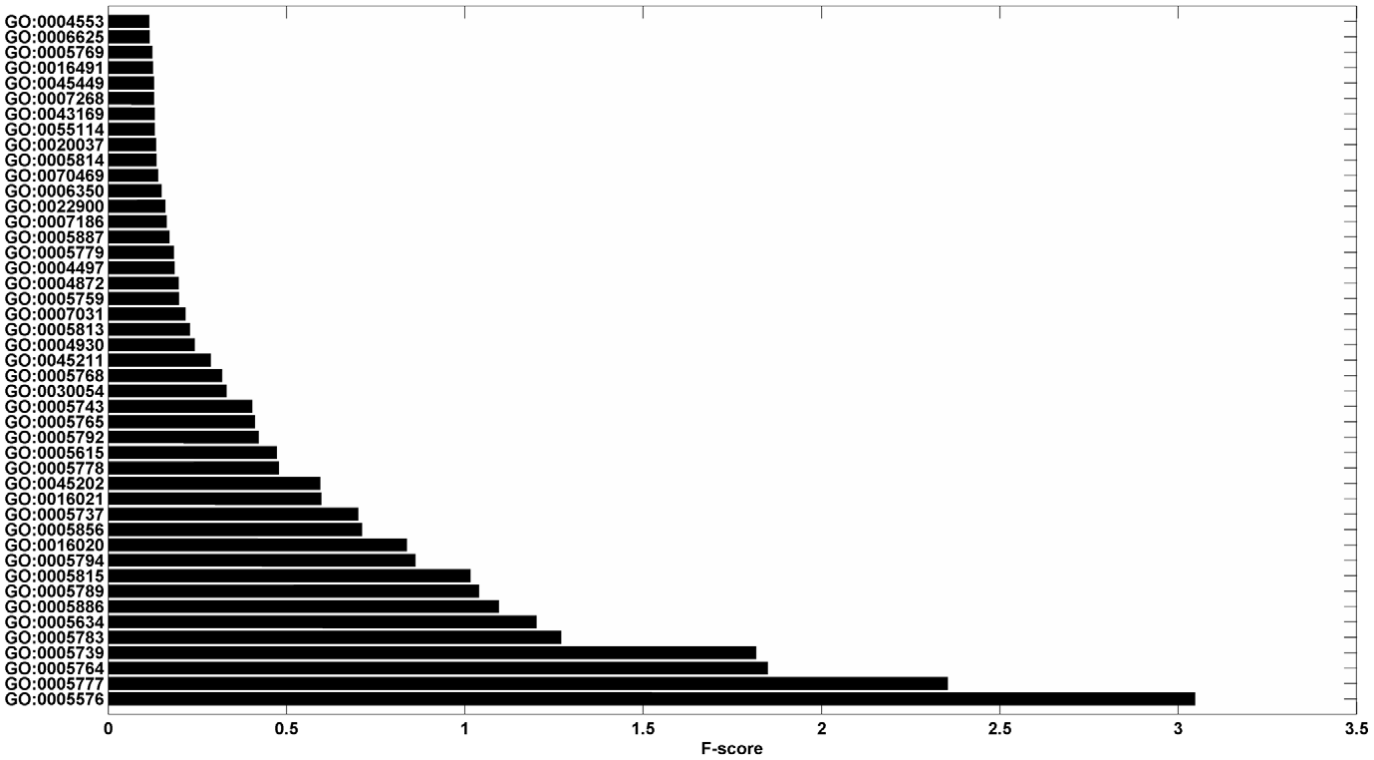
This graph shows the contribution scores of top 45 features on the Hum3681 dataset.

### 5. The KNN-SVM ensemble classifier

**Figure 4:**
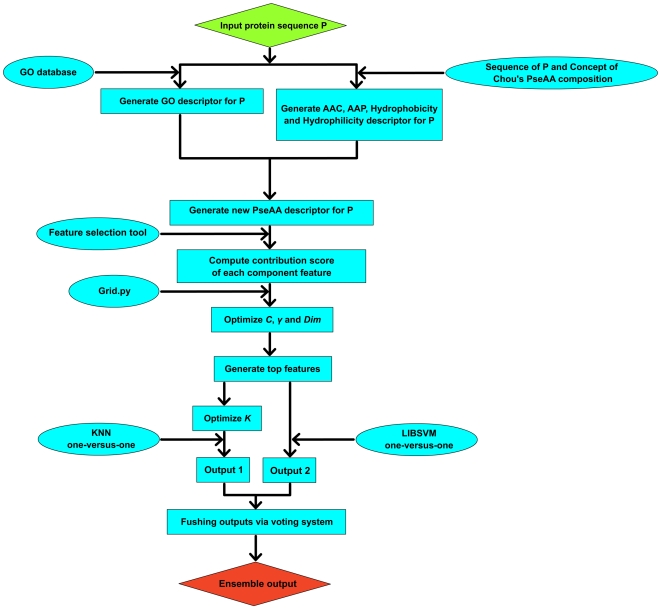
This graph shows the flow chart for application of KNN and LIBSVM algorithms.

A wide variety of machine learning methods have been proposed for predicting protein subcellular localization in recent years [47], [48], [49], [50], such as Markov chain models [51], neural networks [46], *K*-Nearest Neighborhood (KNN) [18], and Support Vector Machines (SVM) [52], [53]. In these methods, KNN and SVM are two popular classifiers in machine learning task. Previous studies presented that each algorithm has its own advantage and the ensemble classifier of different algorithms is the future direction of protein subcellular localization prediction. So, in this paper we proposed an ensemble classifier of KNN and SVM based on *one-versus-one* strategy and a voting system (**Fig. 4**). LIBSVM still has a few tunable parameters which affect the accuracy of the subcellular localization prediction and need to be determined. In this article, “grid.py” was used in the iLoc8897 dataset to select the parameter 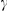 and the regularization parameter 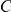 in LIBSVM [24]. Here, the iLoc8897 dataset was selected for optimization of the parameters of the classification models due to the following reasons: (i) compared to the other datasets, this dataset has the largest number of proteins, so it possesses a distinct statistical significance for training; (ii) sequences in this dataset have relatively low pairwise sequence homology; (iii) this dataset covers enough subcellular locations and was widely adopted for evaluating a new proposed method [30], [38].

Prediction of protein subcellular localization is a multi-class classification problem. Here, the class number is equal to 22 for iLoc8897 dataset, 12 for Euk7579 dataset and 14 for Hum3681 dataset, respectively. A simple way to deal with the multi-class classification is to reduce the multi-classification to a series of binary classifications. During this study, we adopted the *one-versus-one* method, i.e., 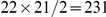, 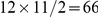, and 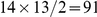 binary classification tasks were constructed for the iLoc8897, Euk7579 and Hum3681 datasets. Compared to the *one-versus-one* approach, the *one-versus-rest* strategy has the shortage that the numbers of positive and negative training data points are not symmetric [54]. For each binary classification, the predictor (KNN or SVM) with the higher output accuracy was selected, and the free parameters, i.e., 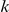 for KNN and 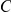 and 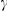 for LIBSVM, are optimized by the iLoc8897 dataset.

Take the Hum3681 dataset as an example. Following the *one-versus-one* strategy, 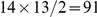 binary classification tasks were constructed for this dataset. For each binary classification task, the KNN and SVM are used to predict the attribute of each protein. As a result, we chose the predictor with the higher output accuracy, where the parameters of KNN and SVM were optimized by the iLoc8897 dataset. Then a score function was generated by the KNN-SVM ensemble classifier formed by fusing the 91 individual binary classifiers through a voting system (see **Eqs. 1** **–** **3**). Each protein was assigned to the subcellular location where the score function has the maximum value. Suppose that the predicted classification results for the query human protein 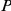 for the 91 binary classifiers are 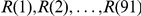, that is
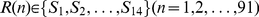
(1)where 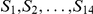 represent the 14 subcellular locations. The voting score for the protein 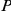 belonging to class 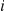 is defined as
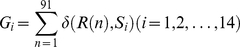
(2)where the 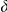 function in **Eq. 2** is given by
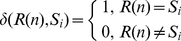
(3)


Subsequently, the query protein 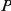 was assigned to the class that gives the highest score for **Eq. 2** of the 91 binary classifiers. We can assume that there are five subsets and 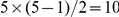 binary classification tasks are constructed. If the predicted classification results for a query protein 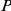 with the ten binary classifiers are 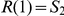, 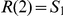, 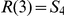, 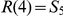, 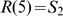, 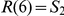, 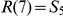, 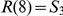, 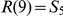, 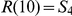 that is, classifiers 1, 2, 3, 4, 5, 6, 7, 8, 9 and 10 assign protein 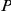 to subsets 2, 1, 4, 5, 2, 2, 5, 3, 5 and 4, respectively. As a result, the voting scores for protein 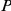 are 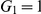, 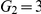, 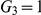, 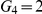, 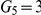. Then protein 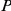 was predicted to classes 2 and 5, which both give the highest score of 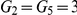.

### 6. Assessment of prediction performances

The prediction quality is examined by the jackknife test currently. Three methods, i.e., the jackknife test, sub-sampling test, and independent dataset test are often used for examining the accuracy of a statistical prediction method. The jackknife test is deemed the most objective and rigorous one [55], [56].

The accuracy, the overall accuracy, the “absolute true” overall accuracy and Matthew's Correlation Coefficient (MCC) [57] for each subcellular location calculated for assessment of the prediction system are formulated as
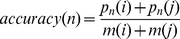
(4)

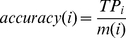
(5)

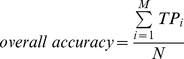
(6)

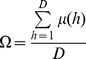
(7)

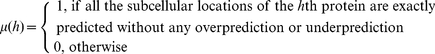
(8)

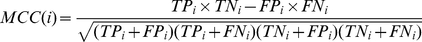
(9)where 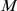 is the class number, 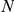 is the total number of locative proteins, 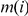 and 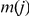 are the numbers of the locative proteins in classes 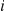 and 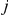, 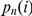 and 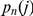 are the numbers of the correctly predicted locative proteins of class 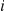 and class 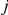 by binary classifier 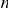. 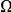 is the so-called “absolute true” overall accuracy. 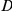 is the number of total proteins investigated. 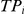, 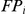, 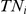, and 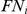 are the numbers of true positives, false positives, true negatives, and false negatives in class 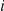 by the KNN-SVM ensemble classifier, respectively.

## Results and Discussion

### 1. Selection of algorithms and parameters

It is important to point out that the best combination of parameters 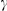 and 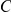 depends on the dimension 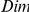 of the protein top feature vector. In the present work, we select the parameters 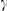 and 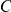 when parameter 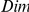 varied from 10 to 50. As seen in **Table 2**, the highest prediction accuracy was 78.01% at 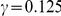, 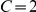 and 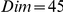. While the prediction accuracy obtained by KNN changed as parameter 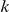 varied from 1 to 9, and the highest prediction accuracy (74.70%) was obtained at 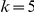 and 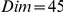 for the iLoc8897 dataset. Then the same parameters, i.e., 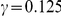, 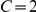, 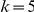 and 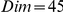 were used for all the three datasets.

**Table 2:** Prediction performance of different top-*N* features on the iLoc8897 dataset by LIBSVM.

	Top10	Top15	Top20	Top25	Top30	Top35	Top40	Top45	Top50
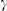	0.03125	0.5	0.5	0.125	0.125	0.125	0.125	0.125	0.125
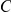	512	0.03125	0.03125	2	2	2	2	2	2
Overall accuracy (%)	51.14	73.08	75.12	74.18	74.40	77.46	77.65	78.01	77.98
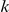	-	-	-	-	-	-	-	5	-
Overall accuracy (%)	-	-	-	-	-	-	-	74.70	-

Because the Hum3681 dataset has 14 subcellular locations, a total of 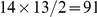 binary classification tasks were constructed. For each *one-versus-one* classification task, the algorithm (KNN or SVM), which gave a higher prediction accuracy for **Eq. 4**, was adopt as the final classifier. For example, the 6^th^, 21^st^, 26^th^, 32^nd^, 34^th^, 42^nd^, 43^rd^, 76^th^, 82^nd^, 84^th^ and 90^th^ binary classifiers (11 of 91 classifiers) was based on the KNN method, because the accuracy of KNN method was higher than LIBSVM method by jackknife test, while the rest 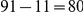 binary classifiers were based on LIBSVM, because the accuracy of LIBSVM method was higher than KNN method by jackknife test.

In addition, most of the existing methods for predicting protein subcellular localization are limited to a single location. It is instructive to note that the KNN-SVM ensemble classifier can effectively deal with multiple-location proteins as well, that is, the predicted result for a query protein 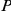 may be attributed to two or more subcellular locations. For example, the real subcellular locations of the protein entry “Q05329” in iLoc8897 dataset are 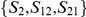, and the predicted subcellular locations for “Q05329” by the KNN-SVM ensemble classifier are also 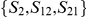, because 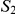, 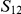, 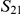 give the highest score (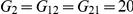) according to **Eq. 2**.

### 2. Comparison with other methods

In order to check the performance of our method, we made comparisons with the following methods: iLoc-Euk [30], Euk-mPLoc 2.0 [38], Hum-mPLoc 2.0 [31], LOCSVMPSI [58], Complexity-based method [59], and the method proposed by Park and Kanehisa [8] which are also based on the Euk7579 dataset. We also compared our method with the KNN binary classifiers, LIBSVM binary calssifiers, and the KNN-SVM ensemble classifier [25]. The comparison is summarized in **Tables 3** **,** **4** **,** **5** **, and** **6**.

**Table 3:** Performance comparisons for eukaryotic protein subcellular location prediction method based on the iLoc8897 dataset.

Subcellular location	Euk-mPLoc 2.0 (2010) (Chou and Shen 2010)	iLoc-Euk (2011) (Chou et al. 2011)	LIBSVM		KNN		The proposed method	
	Jackknife	Jackknife	Jackknife		Jackknife		Jackknife	
	Accuracy (%)	Accuracy (%)	Accuracy (%)	MCC	Accuracy (%)	MCC	Accuracy (%)	MCC
Acrosome	7.14	7.14	57.14	0.8526	71.43	0.8449	64.29	0.8659
Cell membrane	64.85	80.49	84.52	0.9123	96.67	0.8558	85.09	0.9121
Cell wall	12.24	16.33	91.84	0.8750	85.71	0.8981	91.84	0.8750
Centrosome	22.92	69.79	86.17	0.8650	92.55	0.6513	88.30	0.8688
Chloroplast	82.60	87.79	99.73	0.9943	99.73	0.9873	99.73	0.9943
Cyanelle	59.49	64.56	100.00	1.0000	98.73	1.0000	100.00	1.0000
Cytoplasm	64.87	76.72	45.24	0.9399	90.34	0.8198	45.70	0.9361
Cytoskeleton	31.65	27.34	50.36	0.7629	6.47	0.8318	49.64	0.7640
Endoplasmic reticulum	76.15	89.06	87.72	0.9529	84.65	0.9457	87.72	0.9542
Endosome	4.88	7.32	21.95	0.7272	19.51	0.8163	21.95	0.7497
Extracell	81.87	90.46	91.82	0.9812	88.64	0.9902	91.92	0.9824
Golgi apparatus	22.05	63.39	76.59	0.8997	46.83	0.9633	77.38	0.9131
Hydrogenosome	20.00	0.00	100.00	1.0000	70.00	1.0000	100.00	1.0000
Lysosome	45.61	31.58	87.72	0.8813	57.89	0.9851	87.72	0.8813
Melanosome	0.00	2.13	76.60	0.9474	14.89	1.0000	76.60	0.9474
Microsome	7.69	0.00	69.23	0.8579	15.38	1.0000	69.23	0.8579
Mitochondrion	70.00	77.05	78.03	0.9749	80.66	0.9688	78.20	0.9750
Nucleus	64.70	87.93	93.69	0.8865	50.65	0.9943	93.60	0.8873
Peroxisome	50.91	54.55	100.00	0.9650	74.55	1.0000	100.00	0.9650
Spindle pole body	33.82	66.18	95.59	0.9110	4.41	1.0000	95.59	0.9181
Synapse	0.00	38.30	80.85	0.7918	25.53	0.8399	80.85	0.7918
Vacuole	59.41	71.76	95.88	0.9399	80.59	0.9819	93.53	0.9606
Overall accuracy	64.17	79.06	78.01	-	74.70	-	78.17	-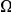	-	71.27	75.54	-	72.84	-	75.64	-

**Table 4:** Performance comparisons for eukaryotic protein subcellular location prediction method based on the Euk7579 dataset.

Subcellular location	Park et al. (2003) (Park and Kanehisa 2003)		LOCSVMPSI (2005) (Xie et al. 2005)	Complexity-based method (2009) (Zheng et al. 2009)	LIBSVM		KNN		The proposed method	
	Jackknife	5-Fold cross	5-Fold cross	Jackknife	Jackknife		Jackknife		Jackknife	
	Accuracy (%)	Accuracy (%)	Accuracy (%)	Accuracy (%)	Accuracy (%)	MCC	Accuracy (%)	MCC	Accuracy (%)	MCC
Chloroplast	57	72.3	76.5	86.4	93.21	0.9982	85.52	0.9689	93.21	0.9982
Cytoplasm	88	72.2	76.4	81.6	87.81	0.9035	89.13	0.7444	87.81	0.9013
Cytoskeleton	44	58.5	60.0	77.5	12.82	1.0000	35.90	0.9660	35.90	0.9660
Endoplasmic reticulum	31	46.5	61.4	78.9	59.82	0.9708	27.68	0.9276	59.82	0.9708
Extracell	57	78.0	89.7	84.0	91.01	0.9746	85.92	0.8879	91.01	0.9739
Golgi apparatus	12	14.6	46.8	61.7	33.33	1.0000	22.22	0.9127	33.33	0.9682
Lysosomal	54	61.8	62.4	73.1	67.74	0.9691	16.13	0.9392	67.74	0.9691
Mitochondrion	42	57.4	68.2	62.9	87.02	0.9502	70.99	0.9017	87.15	0.9494
Nucleus	73	89.6	91.5	84.4	95.94	0.8710	81.85	0.9441	95.94	0.8741
Peroxisomal	4	25.2	41.6	62.4	66.94	0.9648	20.16	0.8446	66.94	0.9648
Plasma membrane	91	92.2	94.7	86.7	93.07	0.9647	93.98	0.9140	93.07	0.9647
Vacuolar	25	25.0	40.7	66.7	50.94	0.9648	0.00	-	50.94	0.9330
Overall accuracy	75	78.2	83.5	81.6	89.80	-	81.60	-	89.94	-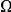	-	-	-	-	89.65	-	81.60	-	89.73	-

**Table 5:** Performance comparisons for human protein subcellular location prediction method based on the Hum3681 dataset.

Subcellular location	Hum-mPLoc 2.0 (2009) (Shen and Chou 2009)	LIBSVM		KNN		The proposed method	
	Jackknife	Jackknife		Jackknife		Jackknife	
	Accuracy (%)	Accuracy (%)	MCC	Accuracy (%)	MCC	Accuracy (%)	MCC
Centriole	-	93.51	0.9240	93.51	0.8867	94.81	0.9249
Cytoplasm	-	39.66	0.9151	91.43	0.7218	41.37	0.9007
Cytoskeleton	-	51.90	0.8138	8.86	0.8816	51.90	0.8232
Endosome	-	54.17	0.7012	33.33	0.7552	54.17	0.7417
Endoplasmic reticulum	-	78.85	0.9046	79.30	0.8960	78.85	0.9043
Extracell	-	86.23	0.9705	82.60	0.9029	86.23	0.9689
Golgi apparatus	-	70.19	0.8853	39.75	0.9284	70.19	0.8887
Lysosome	-	93.51	0.9407	57.14	0.9777	93.51	0.9407
Microsome	-	50.00	0.8008	0.00	-	50.00	0.8008
Mitochondrion	-	84.89	0.9569	81.04	0.9763	83.79	0.9596
Nucleus	-	91.67	0.8876	50.15	0.9833	91.77	0.8932
Peroxisome	-	97.87	0.9380	51.06	0.9605	97.87	0.9481
Plasma membrane	-	84.66	0.8887	60.80	0.9618	84.66	0.8870
Synapse	-	86.36	0.8487	27.27	0.8657	86.36	0.8487
Overall accuracy	62.7	75.22	-	67.75	-	75.55	-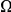	-	72.22	-	65.19	-	72.25	-

**Table 6:** Performance comparisons for eukaryotic protein subcellular location prediction method based on the Euk6181 dataset.

Subcellular location	Euk-mPloc	KNN-SVM ensemble classifier (2010)				The proposed method	
	Jackknife	Jackknife		Resubstitution		Jackknife	
	Accuracy(%)	Accuracy(%)	MCC	Accuracy(%)	MCC	Accuracy(%)	MCC
Acrosome	-	41.2	0.641	76.5	0.874	76.47	0.9308
Cell wall	-	67.9	0.711	88.7	0.903	92.45	0.9028
Centriole	-	62.5	0.690	81.3	0.786	89.06	0.8857
Chloroplast	-	97.4	0.879	99.0	0.918	97.80	0.9956
Cyanelle	-	91.8	0.957	91.8	0.957	100.00	1.0000
Cytoplasm	-	88.2	0.640	91.8	0.729	82.64	0.7946
Cytoskeleton	-	24.3	0.491	41.9	0.645	0.00	0.0000
Endoplasmic reticulum	-	79.7	0.776	86.8	0.839	77.20	0.8906
Endosome	-	62.9	0.770	67.4	0.812	65.17	0.7867
Golgi apparatus	-	74.0	0.802	79.5	0.828	81.89	0.8355
Hydrogenosome	-	38.5	0.620	69.2	0.692	100.00	1.0000
Lysosome	-	65.0	0.662	72.5	0.772	98.75	0.9106
Melanosome	-	53.9	0.733	84.6	0.880	76.92	1.0000
Microsome	-	19.4	0.380	41.9	0.647	9.68	0.5996
Mitochondrion	-	85.1	0.872	87.5	0.910	89.91	0.9425
Nucleus	-	84.6	0.824	85.7	0.862	61.97	0.9642
Peroxisome	-	37.1	0.589	74.2	0.860	98.97	0.9896
Plasma membrane	-	81.4	0.766	84.4	0.817	71.86	0.9373
Extracell	-	83.3	0.864	85.9	0.894	92.81	0.9537
Spindle pole body	-	50.0	0.669	75.0	0.850	72.22	0.8679
Synapse	-	66.7	0.816	66.7	0.816	53.33	1.0000
Vacuole	-	42.2	0.610	82.4	0.865	92.16	0.9181
Overall accuracy	67.4	70.5	-	77.6	-	79.14	-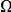	-	-	-	-	-	77.62	-

For the iLoc8897 dataset, the absolute true overall accuracy of the current approach is 75.64%, which is 4.37% higher than the iLoc-Euk method, though the overall accuracy is only 0.89% lower than it. In addition, our method achieves the best performances among the 22 subcellular locations except for the locations of Cytoplasm and Endoplasmic reticulum. Meanwhile, our method also performs better than Euk-mPLoc 2.0 [38] which is also based on the same dataset. For the Euk7579 dataset, the overall accuracy of the current approach is 89.94%, which is also higher than those achieved using the methods listed in **Table 4** (from 6.44% to 14.94%). Meanwhile, our method also performs better than some other classifiers such as LOCSVMPSI [58] and complexity-based method [59]. As shown in **Table 5**, our method also achieves better performances than Hum-mPLoc 2.0. For the Hum3681 dataset, the overall accuracy of the current approach is 75.55%, which is 12.85% higher than the Hum-mPLoc 2.0 method. It is worth noting that all the three datasets (Euk-mPLoc 2.0, iLoc-Euk and Hum-mPLoc 2.0), which also extract sequence features from the Gene Ontology information to represent the query protein, get the comparable accuracies to the present method. This demonstrates that the Gene Ontology information provides a better source of information for the prediction of protein subcellular location. As shown in **Table 6**, the proposed method, examined by the jackknife test, also performs better than Euk-mPLoc and the KNN-SVM ensemble classifier [25]. For the Euk6181 dataset [60], the overall accuracy of the proposed method is 79.14%, which is 11.74% and 8.64% higher than Euk-mPLoc and the KNN-SVM ensemble classifier respectively [25].

As illustrated by some researchers, protein sequence similarity within the datasets has a significant effect on the prediction performance of protein subcellular location, i.e., accuracies will be overestimated when using high-similarity datasets. To avoid this problem, two low-similarity datasets, i.e., the iLoc8897 dataset and Hum3681 dataset were used to evaluate the performance of our method. The results also show that our method achieves good performances and the prediction accuracies are higher than those achieved using the methods listed in **Table 3** and **Table 5**.

### 3. A case study

To evaluate the performance of the proposed method, it was also used to predict the subcellular locations of some proteins used in our laboratory. Take two proteins for example. The first example is fibronectin (FN) [61], [62], which is an “extracell” protein and abundant in the extracellular matrix and participates in many cellular processes, including osteoblastic differentiation/mineralization, tissue repair, embryogenesis, cell migration/adhesion, and blood clotting. The accession number for FN is shown in **Table 7**. According to our ensemble classifier, this protein was predicted as “extracell” protein, which is in accordance with the annotation in Swiss-Prot database. The second is cadherin 11 (CDH 11) [61], [62], which is a plasma membrane protein preferentially expressed in osteoblasts. CDH 11 can promote cells to form specialized cell junctions and enhanced crosstalk between adjacent osteocytes. The accession number for CDH 11 is also shown in **Table 7**. We also predicted it correctly. More examples are list in **Table 7**. As is shown, 10 of all the 11 proteins are predicted in accordance with the Swiss-Prot annotations by the proposed method. While only 8 of 11 eukaryotic proteins and 2 of 4 human proteins are predicted correctly by iLoc-Euk and Hum-mPLoc2.0 respectively.

**Table 7:** Examples to show the predicted results by three predictors.

Accession number	Entry name	Swiss-Prot annotation	iLoc-Euk (2011)	Hum-mPLoc 2.0 (2009)	The proposed method
					Trained by iLoc8897 dataset
P55287	Cad11_human	Plasma membrane	Plasma membrane	Plasma membraneCytoplasmExtracell	Plasma membrane
P02751	Finc_human	Extracell	Extracell	Extracell	Extracell
Q8IZC6	Cora1_human	Extracell	Extracell		Extracell
Q9EPU7	Z354c_rat	Nucleus	Nucleus	-	Nucleus
Q5QNQ9	Cora1_mouse	Extracell	Extracell	-	Extracell
Q5BKR2	Nhdc2_mouse	Mitochondrion	Plasma membrane	-	Mitochondrion
P12645	Bmp3_human	Extracell	Extracell	Extracell	Extracell
P51690	Arse_human	Golgi apparatus	Cytoplasm	Lysosome	Golgi apparatus
Q8C341	Ospt_mouse	Endoplasmic reticulum	Plasma membrane	-	Cytoplasm
P00922	Cah2_sheep	Cytoplasm	Cytoplasm	-	Cytoplasm
Q30D77	Cooa1_mouse	Extracell	Extracell	-	Extracell

We also used iLoc-Euk, Hum-mPLoc 2.0 and the proposed method to predict the subcellular locations of some multiple-location proteins. As can be seen from **Table 8**, all subcellular locations of the protein Q05329 was correctly identified by the proposed method and iLoc-Euk, but not entirely correctly by Hum-mPLoc 2.0. The second protein P58335 was identified completely correctly by the proposed method, but according to iLoc-Euk and Hum-mPLoc 2.0, it was assigned to only one of its real subcellular locations. The third protein P30622 simultaneously exists at “Cytoplasm” and “Cytoskeleton” in Swiss-Prot. Both iLoc-Euk and Hum-mPLoc 2.0 only identified one location correctly. Although the proposed method incorrectly predicted P30622 as belonging to “endosome”, yet it successfully identified two of its subcellular locations.

**Table 8:** Examples to show the predicted results by three predictors on multiple-location proteins.

Accession number	Entry name	Swiss-Prot annotation	iLoc-Euk (2011)	Hum-mPLoc 2.0 (2009)	The proposed method
					Trained by iLoc8897 dataset
Q05329	DCE2_human	Plasma membraneGolgi apparatusSynapse	Plasma membraneGolgi apparatusSynapse	CytoplasmMitochondrionSynapse	Plasma membraneGolgi apparatusSynapse
P58335	Antr2_human	Endoplasmic reticulumPlasma membraneExtracell	Extracell	Endoplasmic reticulum	Endoplasmic reticulumPlasma membraneExtracell
P30622	Clip1_human	CytoplasmCytoskeleton	Cytoplasm	CytoskeletonEndosome	CytoplasmCytoskeletonEndosome
P13395	Sptca_drome	CytoskeletonGolgi apparatusPlasma membrane	Golgi apparatus	-	CytoskeletonGolgi apparatus
P11279	Lamp1_human	EndosomeLysosomePlasma membrane	Plasma membrane	Lysosome	Plasma membraneLysosomeMelanosome
Q15942	Zyx_human	CytoplasmCytoskeleton	Cytoskeleton	Plasma membrane	CytoplasmCytoskeletonNucleus

### 4. Conclusions

In this study, a KNN-SVM ensemble classifier by fusing the GO attributes and hydrophobicity features was investigated to predict subcellular location of eukaryotic proteins. Three widely used benchmark datasets were adopted in our work. To improve the prediction quality, the following strategies were applied: (i) representing protein samples by using Gene Ontology could effectively grasp the core features to indicate the subcellular localization, (ii) adopting the *one-versus-one* strategy and two most popular classifiers in machine learning task, i.e., LIBSVM and KNN to predict protein subcellular location, (iii) capturing the top features and learning with a small number of features might lead to a better generalization of machine learning algorithms (Occam's razor). In summary, the results of the predictions performed by KNN-SVM ensemble classifier indicate that our method is very promising and may play an important complementary role to existing methods.
